# Risk factors associated with chronic cutaneous graft-versus-host disease following hematopoietic stem cell transplantation: a pediatric cohort^؆^

**DOI:** 10.1016/j.abd.2026.501296

**Published:** 2026-03-23

**Authors:** Daniela Carvajal, Teresa Dossi, Camila Downey, Daniela Krämer, Natalia González, Javier Fernández, Paula Muñoz

**Affiliations:** a Department of Pediatric Dermatology, Hospital de Niños Luis Calvo Mackenna, Santiago, Chile; b Department of Oncology, Hospital de Niños Luis Calvo Mackenna, Santiago, Chile; c Clínica Santa María, Santiago, Chile; d Dermatology Department, Universidad de Los Andes, Santiago, Chile

**Keywords:** Child, Dermatology, Graft vs. host disease, Hematopoietic stem cell transplantation, Skin

## Abstract

**Background:**

Graft-versus-host disease (GVHD) is a frequent complication of hematopoietic stem cell transplantation (HSCT), with the skin being the most commonly affected organ. However, its risk factors remain poorly defined.

**Objective:**

To assess the risk factors associated with chronic cutaneous GVHD (ccGVHD) in a children’s hospital in Chile.

**Methods:**

This retrospective cohort study examined children under 18-years who underwent HSCT between 2007 and 2017, with a follow-up period of at least 2-years. Survival analysis and Cox regression analysis were performed.

**Results:**

150 children with HSCT were included with a median age of 7.3 (3.7‒10.6) years. 17.3% of the children developed ccGVHD, with a median onset after HSCT of 8 (4‒11) months. In the univariate analysis, patients with ccGVHD were significantly older at transplantation, with a higher proportion of bone marrow as graft source, related donor, and acute GVHD compared to those who did not develop ccGVHD (for all *p* < 0.05). In the multivariate analysis, the main risk factors were male sex (Hazard Ratio [HR] 2.51), total body irradiation as conditioning regimen (HR = 3.53), and bone marrow as graft source (HR = 7.28).

**Study limitations:**

Its retrospective, single-center design may reduce generalizability and introduce selection bias.

**Conclusions:**

This study is one of the largest series of ccGVHD in children. Early identification of patients at higher risk of ccGVHD allows for timely initiation of treatment, thereby reducing the morbidity associated with this debilitating complication.

## Introduction

Graft-versus-host disease (GVHD) is a significant complication of hematopoietic stem cell transplantation (HSCT), representing a leading cause of both morbidity and mortality.^1^, ^2^ In pediatric patients, HSCT is most commonly performed to treat hematologic malignancies, although its application for non-malignant conditions ‒ including primary immunodeficiencies, bone marrow failure syndromes, and hereditary metabolic disorders ‒ is growing.^3^ Conditioning regimens ‒ administered with or without total-body irradiation (TBI) ‒ are essential to suppress the recipient’s immune system and facilitate engraftment. Donor sources may include family members or unrelated donors, with hematopoietic progenitors derived from bone marrow, umbilical cord blood (UCB), or mobilized peripheral blood stem cells (PBSC).^2^

GVHD is divided into acute and chronic forms, which differ in their clinical presentation, risk factors, and long-term consequences. These two forms can occur independently of each other.^1^ The skin is the most affected organ in both.^4^, ^5^ Distinction between acute and chronic GVHD is defined by specific clinical characteristics.^1^

Manifestations of cutaneous chronic GVHD (ccGVHD) are diverse.^6^ According to the National Institutes of Health (NIH) Consensus Development Project, the presence of one of the following manifestations alone is sufficient to diagnose ccGVHD: poikiloderma, lichen planus-like eruptions, sclerodermoid features, morphea-like features, and lichen sclerosus-like features.^7^

Skin biopsy is indicated when clinical manifestations of ccGVHD are inconclusive. Histopathologically, ccGVHD can be classified into epidermal, sclerotic, or combination disease. Epidermal ccGVHD predominantly shows a lichen planus/interface dermatitis pattern, whereas sclerotic ccGVHD is characterized by dermal and subcutaneous sclerosis with a lichen sclerosus/morphea-like pattern. Minimal pathologic features of active GVHD ‒ including apoptosis in the epidermal basal layer, vacuolar change, and lymphocyte satellitosis ‒ are common in all biopsies; however, most specimens also exhibit findings overlapping with autoimmune and inflammatory skin diseases.^8^

Previous studies in the pediatric population have shown a prevalence of ccGVHD between 18%‒32% in recipients of HSCT.^2^, ^9^ Early diagnosis and treatment of ccGVHD is essential, due to its high morbidity and deterioration in quality of life. Studies in the pediatric population are scarce, with few patients and a retrospective design. The objective of this study is to assess the risk factors of ccGVHD in children undergoing HSCT at a children’s hospital in Chile.

## Methods

This retrospective cohort study included all children under 18-years of age undergoing HSCT between 2007 and 2017 with a clinical follow-up period of at least 2-years, at the Luis Calvo Mackenna Children’s Hospital. This study was approved by the Ethics Committee of Research Protocols of the Faculty of Medicine at the University of Chile, Santiago, Chile (136-2019).

In all patients, the diagnosis of ccGVHD and acGVHD was established by pediatric dermatologists, based on clinical manifestations defined according to the standardized criteria of the NIH Consensus Development Project.^7^ In patients presenting with nonspecific clinical manifestations that complicated the differential diagnosis, a skin biopsy was performed.

Recorded data included patient demographics, diagnoses that required HSCT, conditioning with TBI, and donor characteristics (sex, graft source and type of donor). Presence of ccGVHD and its characteristics: clinical type of ccGVHD, time interval between HSCT and onset of ccGVHD, presence of prior or concomitant acGVHD, lung involvement, and mortality.

The exclusion criterion was a personal history of autoimmune skin disease prior to the transplant.

### Statistical analysis

The inferential analysis was performed by the Wilcoxon rank-sum test and the Fisher exact test. Survival analysis was performed by the Kaplan-Meier method, and cumulative hazard was estimated by the Nelson-Aalen method. Univariate and multivariate Cox regression analysis were used to evaluate the magnitude of association of risk factors, estimating the Hazard Ratio (HR) and the 95% Confidence Interval (95% CI). The proportional hazards assumption of Cox regression was verified. The level of significance was defined as *p* < 0.05. Statistical analysis was performed using the statistical software STATA 14® (StataCorp).

## Results

### Study population

One hundred fifty children with HSCT were included, and no patients undergoing HSCT during the study period were excluded. At the time of transplant, the median and Interquartile Range (IQR) age was 7.3 (3.7‒10.6) years, and 60% of the patients were male (Table 1). All patients undergoing HSCT were followed for at least 24-months, with a median follow-up time of 52 (30‒98) months. The main diagnoses that required HSCT were acute lymphoblastic leukemia in 82 patients (54.7%) and acute myeloid leukemia in 30 patients (20%). The most common graft sources were UCB in 79 patients (52.7%) and bone marrow in 71 patients (47.3%). PBSC were not used in this cohort. The type of donor was related in 37.3%, and their main graft source was bone marrow in 96.4%; while in the unrelated group, the main source was UCB in 81.9%. Eleven patients (7.3%) died during the follow-up period, with a median age of 10.3 (6‒16.6) years. The median time from transplantation to death was 4.8 (2.4‒5.9) years.

**Table 1:** Clinical characteristics of children undergoing HSCT with and without ccGVHD.

	All HSCT Patients (*n* = 150)	Patients with ccGVHD (*n* = 26)	Patients without ccGVHD (*n* = 124)	*p*
Age (years, median [IQR])^a^	7.3 [3.7‒10.6]	10 [7‒13]	7 [3.7‒10.3]	**0.013**
Male/female (%)	60 / 40	73.1 / 26.9	57.3 / 42.7	0.186
Diagnosis				0.545
Acute myeloid leukemia (%)	20	11.5	21.8	
Lymphoblastic leukemia (%)	54.7	61.5	53.2	
Others (%)	25.3	27	25	
Total-body irradiation (%)	44.7	57.7	41.9	0.193
Sex of the donor (Female, %)	47.7	57.7	45.5	0.286
Graft source				**0.001**
Umbilical cord (%)	52.7	23.1	58.9	
Bone marrow (%)	47.3	76.9	41.1	
Type of donor				**0.002**
Unrelated (%)	62.7	34.6	68.5	
Related (%)	37.3	65.4	31.5	
Acute cutaneous GVHD (%)	62	80.8	58.1	**0.044**
Lung involvement (%)	6	11.5	4.8	0.189
Mortality (%)	7.3	7.7	7.3	1.000

HSCT, Hematopoietic Stem Cell Transplantation; ccGVHD, Chronic cutaneous Graft-Versus-Host Disease; IQR, Interquartile range.

Values in bold indicate statistical significance (*p* < 0.05).

a Age at transplantation.

### Cutaneous chronic graft-versus-host disease characteristics

**Fig. 1:**
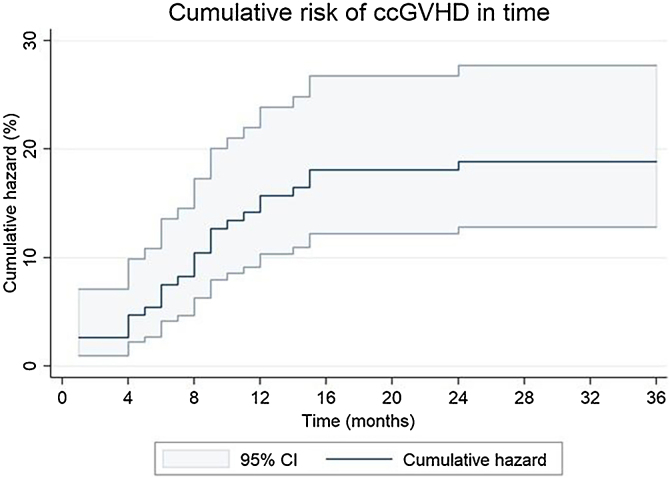
Nelson-Aalen estimator of cumulative risk of ccGVHD with 95% CI. CcGVHD, Chronic cutaneous Graft-Versus-Host Disease; CI, Confidence Interval.

The cumulative incidence of ccGVHD over a 36-month period was 17.3% (95% CI: 12.2%‒24.4%) in this cohort. Patients with ccGVHD had a median age at transplantation of 10 (7‒13) years, and 73.1% were male. The median onset of ccGVHD following HSCT was 8 (4‒11) months. The cumulative risk over time increased steadily between 4- and 15-months, and then remained stable (Fig. 1).

**Fig. 2:**
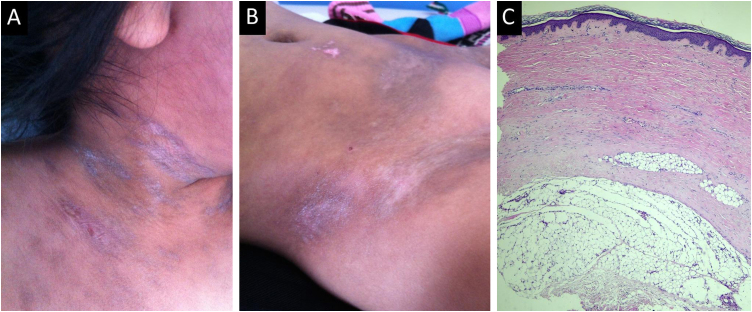
Female patient with cutaneous chronic graft-versus-host disease. (A) Sclerodermoid plaques involving the neck and upper chest. (B) Lesions affecting the skin of the lower abdomen, hip, and inguinal fold. (C) Skin biopsy showing epidermal atrophy with focal vacuolar degeneration of the dermal-epidermal junction. Marked fibrosis composed of parallel bundles of collagen in the superficial and deep dermis and subcutaneous tissue, with atrophy of cutaneous adnexal structures (Hematoxylin & eosin, ×10).

**Fig. 3:**
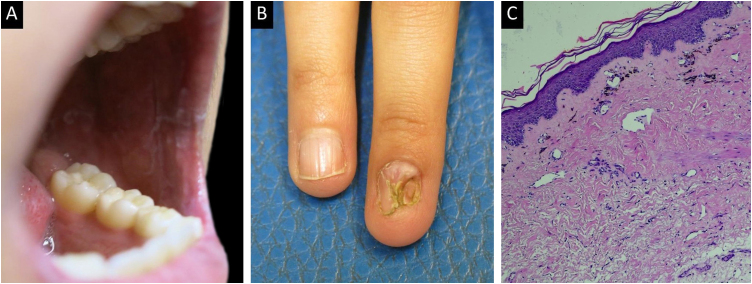
Female patient with lichenoid manifestations of cutaneous chronic graft-versus-host disease. (A) White reticulations on the buccal mucosa. (B) Nail changes with longitudinal ridging and pterygium formation. (C) Biopsy showing skin lined by a thin orthokeratotic epidermis with focal vacuolar degeneration of the dermal-epidermal junction and occasional dyskeratotic keratinocytes, accompanied by intraepithelial lymphocytes. The superficial dermis shows telangiectatic vessels, fibrosis, and numerous melanophages, with a reduction in cutaneous adnexal structures (Hematoxylin & eosin, ×10).

Cutaneous manifestations of ccGVHD, which may occur independently or simultaneously, included sclerodermoid features in 30.4%, lichenoid features in 26.1%, combined sclerodermoid and lichenoid features in 17.4%, eczema-like lesions in 13%, vitiligo-like depigmentation in 8.7%, and poikiloderma in 4.4% of patients. Skin biopsy was performed in 61.5% (*n* = 16) of cases. Representative clinical presentations together with their corresponding histopathological features are shown in Fig. 2, Fig. 3.

Patients with ccGVHD, compared to patients without ccGVHD, were significantly older at transplantation (Table 1). The group with ccGVHD had a higher percentage of bone marrow source and related donor compared with patients without ccGVHD. The incidence of prior or concomitant acGVHD was significantly higher in patients with ccGVHD compared with patients without ccGVHD.

Univariate analysis (Table 2) revealed that age at transplantation presented a HR of 1.008 (95% CI: 1.001‒1.015), bone marrow source compared to UCB a HR of 4.29 (95% CI: 1.72‒10.69), related compared to unrelated type of donor a HR of 3.58 (95% CI: 1.59‒8.03) and history of acGVHD a HR of 2.76 (95% CI: 1.04‒7.34) for developing ccGVHD.

**Table 2:** Univariate Cox regression analysis of ccGVHD risk factors.

	Univariate Analysis		
	Hazard Ratio	95% CI	*p*
Age (years)^a^	1.008	1.001 ‒ 1.015	**0.011**
Sex (Male)	1.86	0.78 ‒ 4.44	0.158
Diagnosis			
Acute myeloid leukemia	1	‒	‒
Lymphoblastic leukemia	2.04	0.59 ‒ 7.03	0.254
Others	1.87	0.48 ‒ 7.23	0.364
Total-body irradiation	1.79	0.82 ‒ 3.90	0.142
Sex of the donor (Female)	1.51	0.69 ‒ 3.30	0.293
Graft source			
Umbilical cord	1	‒	‒
Bone marrow	4.29	1.72 ‒ 10.69	**0.002**
Type of donor			
Unrelated	1	‒	‒
Related	3.58	1.59 ‒ 8.03	**0.002**
Acute cutaneous GVHD	2.76	1.04 ‒ 7.34	**0.041**
Lung involvement	2.3	0.69 ‒ 7.69	0.173

ccGVHD, Chronic cutaneous Graft-Versus-Host Disease; CI, Confidence Interval.

Values in bold indicate statistical significance (*p* < 0.05).

a Age at transplantation.

Multivariate analysis (Table 3) demonstrated that male sex presented a HR of 2.51 (95% CI: 1.04‒6.06), TBI as conditioning regimen a HR of 3.53 (95% CI: 1.55‒8.03), and bone marrow source compared to UCB a HR of 7.28 (95% CI: 2.77‒19.10) for developing ccGVHD.

**Table 3:** Multivariate Cox regression analysis of ccGVHD risk factors.

	Multivariate Analysis		
	Hazard Ratio	95% CI	*p*
Sex (Male)	2.51	1.04 ‒ 6.06	**0.040**
Total-body irradiation	3.53	1.55 ‒ 8.03	**0.003**
Graft source			
Umbilical Cord	1	‒	‒
Bone Marrow	7.28	2.77 ‒ 19.10	**0.001**

ccGVHD, Chronic cutaneous Graft-Versus-Host Disease; CI, Confidence Interval.

Values in bold indicate statistical significance (*p* < 0.05).

Patients who developed ccGVHD did not have a difference in mortality compared to patients without ccGVHD, with a HR of 0.78 (95% CI: 0.16‒3.64, *p* = 0.756).

## Discussion

This study represents a large series of ccGVHD in children in Chile. In our study, 17.3% of the children developed ccGVHD, with a median onset of 8-months. Based on these findings, we recommend active surveillance for ccGVHD for at least 15-months following transplantation. An Israeli study of 112 children undergoing HSCT showed an incidence of ccGVHD of 18%, with a mean time interval from transplantation of 4.9 ± 3.95 months.^2^ A publication from Kuwait in 14-patients showed a median time between HSCT and onset of ccGVHD of 7.5-months.^10^ An Argentinian study in patients under 20-years of age showed an incidence of ccGVHD of 32%.^9^ These differences in incidence can be explained by differences in protocols, graft sources, and the type of donors used in each reference center.^2^, ^9^

Alvarado et al. conducted a histopathological analysis of ccGVHD in 49 adult patients. Clinically, 67.3% had epidermal ccGVHD, 2.1% sclerotic ccGVHD, and 30.6% combination disease. Epidermal ccGVHD predominantly showed a lichen planus–like/interface dermatitis pattern with basal keratinocyte apoptosis (94%) and vacuolar alteration (92.5%), whereas sclerotic ccGVHD showed a higher proportion of thickening of the papillary and reticular collagen and the subcutaneous septae (up to 68.8%) with a lichen sclerosus/morphea-like pattern. Subclinical sclerosis was noted in several epidermal ccGVHD lesions, suggesting potential prognostic implications.^8^ In our cohort, the analyzed biopsies showed findings consistent with those reported in this study, highlighting the importance of comprehensive histologic reporting. Additional studies are warranted to better characterize the histopathologic features of ccGVHD in pediatric patients.

Our cohort of patients with ccGVHD was older at HSCT compared to patients without ccGVHD. Similarly, in the Israeli study, the group with ccGVHD had a significantly older age at transplantation than patients without ccGVHD (9.5 ± 5.2 years vs. 5.4 ± 4.8 years).^2^ However, a Spanish study of 50 children with acute and chronic cutaneous GVHD did not find significant differences between the groups with and without ccGVHD.^11^ The higher risk of chronic GVHD in older patients might reflect a role of age-related atrophy of the thymus, which results in a loss of thymic negative selection post HSCT.^12^, ^13^

UCB transplantation has become more prevalent in recent years in the pediatric population, especially when a related matched bone marrow is unavailable.^1^ In our study, the most common graft source was UCB with 52.67%. Research in pediatric patients has found that the UCB source is associated with a lower risk of acute and chronic GVHD in comparison with the bone marrow source.^14^, ^15^ In our study, bone marrow source had a 4.29 times higher risk in the univariate analysis and a 7.28 times higher risk in the multivariate analysis of developing ccGVHD compared to UCB source. In a meta-analysis in 1453 children, the rate of chronic GVHD in the unrelated UCB group was significantly lower than the unrelated bone marrow group (OR = 0.36, *p* < 0.0001).^14^ One possible explanation is that transplanted UCB stem cells are immunologically naive, reducing their capacity to induce chronic GVHD.^14^, ^15^

Patients with ccGVHD had a significantly higher proportion of history of prior or concomitant acGVHD compared to patients without ccGVHD (80.77% vs. 58.06%), with an HR of 2.76 in the univariate analyses. Therefore, patients with acGVHD should be considered at high risk for developing ccGVHD. 66% of patients with ccGVHD had a history of acGVHD in the Spanish study,^11^ 86% in the Kuwait publication,^10^ and 100% in the Argentinian study.^9^ None of these 3 studies compared the frequency of acGVHD in patients with and without ccGVHD. The evidence shows that acute GVHD damages the thymus, resulting in a loss of thymic negative selection, leading to chronic GVHD.^1^, ^13^

In our study, TBI as a conditioning regimen had a 3.53 times higher risk of developing ccGVHD in the multivariate analysis. The Spanish publication showed that children with ccGVHD had a significantly higher proportion of TBI versus patients without ccGVHD (66% vs. 33%).^11^ TBI administered prior to HSCT results in higher toxicity and tissue damage, establishing a pro-inflammatory response, possibly explaining our results.^2^ Therefore, it is pertinent to consider patients who will undergo TBI as being at higher risk of developing ccGVHD.

The related donor had a 3.58 times higher risk of developing ccGVHD than the unrelated donor in univariate analysis. This may be explained by the source of the graft, being 96% from bone marrow in the related group in comparison to 81.9% from UCB in the unrelated group. As previously discussed, bone marrow source is a known risk factor for ccGVHD compared with UCB.^14^, ^15^

Unlike other studies, we did not find any difference in the risk of ccGVHD comparing the sex of the donor, malignant disease as the cause of HSCT, and lung involvement.^2^, ^11^

## Study limitations

One limitation of this study is its retrospective, single-center design, which may limit external validity. Additionally, the diversity of conditioning regimens, GVHD prophylaxis, and treatment protocols introduces heterogeneity that could further compromise generalizability. However, the selected hospital was the only public pediatric HSCT center in the country, serving as the reference institution for nearly 80% of the public health system’s population. This setting enabled comprehensive and consistent monitoring; every patient was followed for at least 24-months, with no exclusions due to insufficient follow-up.

## Conclusion

This study demonstrates a 17.3% incidence of ccGVHD in pediatric recipients of HSCT, with a median onset of 8-months post-transplant. These findings underscore the importance of sustained vigilance, and we therefore recommend standardized monitoring for ccGVHD up to at least 15-months after HSCT. Our results highlight the need for individualized risk stratification and tailored surveillance protocols in pediatric centers performing HSCT.

While several studies exist with larger cohorts ‒ encompassing all age groups or focused specifically on adults ‒ data exclusive to the pediatric population remain limited. This report should be regarded as a preliminary, single-center study and underscores the need for prospective, multicenter collaborations both nationally and internationally. Such efforts would broaden our understanding of the disease’s influencing factors and support the development of future guidelines for prevention and management.

## ORCID IDs

Teresa Dossi: 0009-0008-6838-2881

Camila Downey: 0000-0002-1624-1170

Daniela Krämer: 0000-0003-3022-3187

Natalia González: 0009-0000-8903-3770

Javier Fernández: 0000-0003-0397-2043

Paula Muñoz: 000-0003-2676-7464

## Authors' contributions

Daniela Carvajal: Writing of the manuscript or critical review of important intellectual content; critical review of the literature; final approval of the final version of the manuscript.

Teresa Dossi: Data collection, analysis and interpretation; critical review of the literature; final approval of the final version of the manuscript.

Camila Downey: Data collection, analysis and interpretation; critical review of the literature; final approval of the final version of the manuscript.

Daniela Krämer: Data collection, analysis and interpretation; critical review of the literature; final approval of the final version of the manuscript.

Natalia González: Data collection, analysis and interpretation; critical review of the literature; final approval of the final version of the manuscript.

Javier Fernández: Data collection, or analysis and interpretation of data; statistical analysis; final approval of the final version of the manuscript.

Paula Muñoz: The study concept and design; data collection, or analysis and interpretation of data; data collection, analysis and interpretation; effective participation in the research guidance; critical review of the literature; final approval of the final version of the manuscript.

## Declaration of Generative AI and AI-assisted technologies in the writing process

During the preparation of this work, the author(s) used ChatGPT in order to improve language and readability. After using this tool/service, the author(s) reviewed and edited the content as needed and take (s) full responsibility for the content of the publication.

## Financial support

This research did not receive any specific grant from funding agencies in the public, commercial, or not-for-profit sectors.

## Research data availability

The entire dataset supporting the results of this study was published in this article.

## Conflicts of interest

None declared.
